# Characteristics and outcomes of pediatric testicular yolk Sac tumor

**DOI:** 10.3389/fped.2022.1024906

**Published:** 2022-12-19

**Authors:** Maoxian Li, Jinkui Wang, Jianyu Wang, Deying Zhang, Yi Hua, Feng Liu, Peng Lu, Junhong Liu, Xing Liu, Tao Lin, Guanghui Wei, Dawei He

**Affiliations:** ^1^ Department of Urology, Ministry of Education Key Laboratory of Child Development and Disorders, National Clinical Research Center for Child Health and Disorders, China International Science and Technology Cooperation Base of Child Development and Critical Disorders, Children's Hospital of Chongqing Medical University, Chongqing, China; ^2^ Chongqing Key Laboratory of Pediatrics, Children's Hospital of Chongqing Medical University, Chongqing, China; ^3^ Chongqing Higher Institution Engineering Research Center of Children's Medical Big Data Intelligent Application, Children's Hospital of Chongqing Medical University, Chongqing, China

**Keywords:** testicular yolk sac tumor, pediatric, relapse, chemotherapy, prognosis

## Abstract

**Purpose:**

Pediatric testicular yolk sac tumor is a rare malignant germ cell tumor and there is a lack of large clinical studies. The purpose of this study is to summarize the clinical characteristics of pediatric testicular yolk tumor and evaluate the prognostic factors.

**Materials and methods:**

The medical records of children with testicular yolk sac tumor in one pediatric medical centre in China from January 2005 to January 2021 were retrospectively investigated. Data regarding clinical characteristics, treatment and prognosis were collected.

**Results:**

A total of 109 patients with a median diagnosed age of 18 months (range 2–69) were included in this study; of them 100 were diagnosed as stage I, 6 as stage II and 3 as stage IV. All patients underwent radical orchiectomy, and 61 of them underwent postoperative chemotherapy. The mean follow-up time was 61.3 months (range 3–259), during that time, 8 patients experienced relapse. The five-year overall survival was 90.6% (95% CI 84.6%–96.7%). Univariate Cox regression analysis showed that disease stage, relapse, maximum tumor diameter, and alpha-fetoprotein returning to normal within 2 months postoperatively were risk factors for survival (HRs of 25.43, 26.43, 1.48 and 0.08, respectively, *p* < 0.05). Multivariate Cox regression analysis suggested that higher disease stage and relapse were independent adverse factors for survival (HRs of 148.30 and 94.58, respectively, *p* < 0.05).

**Conclusions:**

The prognosis of pediatric testicular yolk sac tumor is generally excellent. A higher disease stage and the occurrence of relapse could predict a poor prognosis. The individualized management of children with testicular yolk sac tumor according to risk classification is feasible.

## Introduction

Pediatric testicular tumors comprise approximately 1%–2% of all pediatric solid tumors, with an annual incidence of 0.2–2 per 100,000 boys (1, 2). Testicular yolk sac tumor (TYST) is the most common malignant germ cell tumor in children, accounting for approximately 70%–80% of testicular malignancies, while the incidence is less than 1% (3).

Previous studies have shown that pediatric patients with TYST mainly present with a painless testicular mass (4, 5), and more than 90% of them have elevated serum alpha-fetoprotein (AFP) levels (5). Scrotum ultrasound, chest computed tomography (CT) and abdominal CT/magnetic resonance imaging (MRI) contribute to preoperative diagnosis (4–6). With high malignancy, an insidious onset and a rapid disease course, TYST seriously affects pediatric health (4, 7). Surgical resection of the primary tumor is critical to the treatment (8). Chemotherapy, retroperitoneal lymph node dissection and targeted therapy of distant metastasis make widely disseminated testicular germ cell tumors treatable (3). With modern multidisciplinary care, the prognosis of TYST is excellent, and the event-free survival is approximately 90% (9).

However, due to the low incidence of TYST, clinical studies are lacking, and the predictive factors of outcome remain unclear. In this study, we retrospectively analysed the clinical data of patients with TYST from the Children's Hospital of Chongqing Medical University, the most famous and national pediatric medical centre in western China, to summarize the clinical characteristics and outcomes of pediatric patients with TYST and evaluate the prognostic factors.

## Materials and methods

### Patients and study design

This study was approved by the Ethical Committee of Chongqing Medical University. The clinical data of children with TYST admitted to Children's Hospital of Chongqing Medical University from January 2005 to January 2021 were retrospectively analysed.

The inclusion criteria were as follows: (1) diagnosis of testicular tumor, (2) TYST made by pathological diagnosis, (3) complete clinical data, and (4) available prognosis. The exclusion criteria were as follows: (1) mixed testicular tumor (yolk sac tumor with teratoma), and (2) surgical treatment in other hospitals. Clinical data (including age at diagnosis, presentation, and AFP tumor marker data), imaging data (scrotal ultrasound, chest CT and abdominal pelvic CT/ultrasound), surgical operation data, chemotherapy data, pathological results and follow-up outcomes were collected. TYST was staged according to the staging system proposed by the Children's Oncology Group (COG) (10).

Close follow-up consisted of physical examination, AFP level measurement, chest x-ray/CT, and scrotal and retroperitoneal ultrasonography every 1–3 months for the first postoperative year, then 6 months in the second year, and once a year thereafter. When relapse was suspected, serum AFP levels were first re-evaluated. If the serum AFP level returned to normal postoperatively, while re-evaluated serum AFP continued to rise with or without metastatic disease, relapse was diagnosed.

### Statistical analysis

Continuous variables (age, tumor diameter and follow-up time) are presented as the mean ± standard deviation, and a paired sample *t*-test was used to compare the groups. Count data are represented as the frequency (%) and chi-square test was used for comparisons between groups. Related factors affecting relapse were analysed by Person correlation analysis. The survival and prognostic factors were analysed by univariate and multivariate Cox proportional regression models. All statistical analyses were performed with SPSS version 25 (SPSS Inc., Chicago, IL., United States). Statistical significance was defined as *P* < 0.05.

## Results

### Clinical features

**Figure 1:**
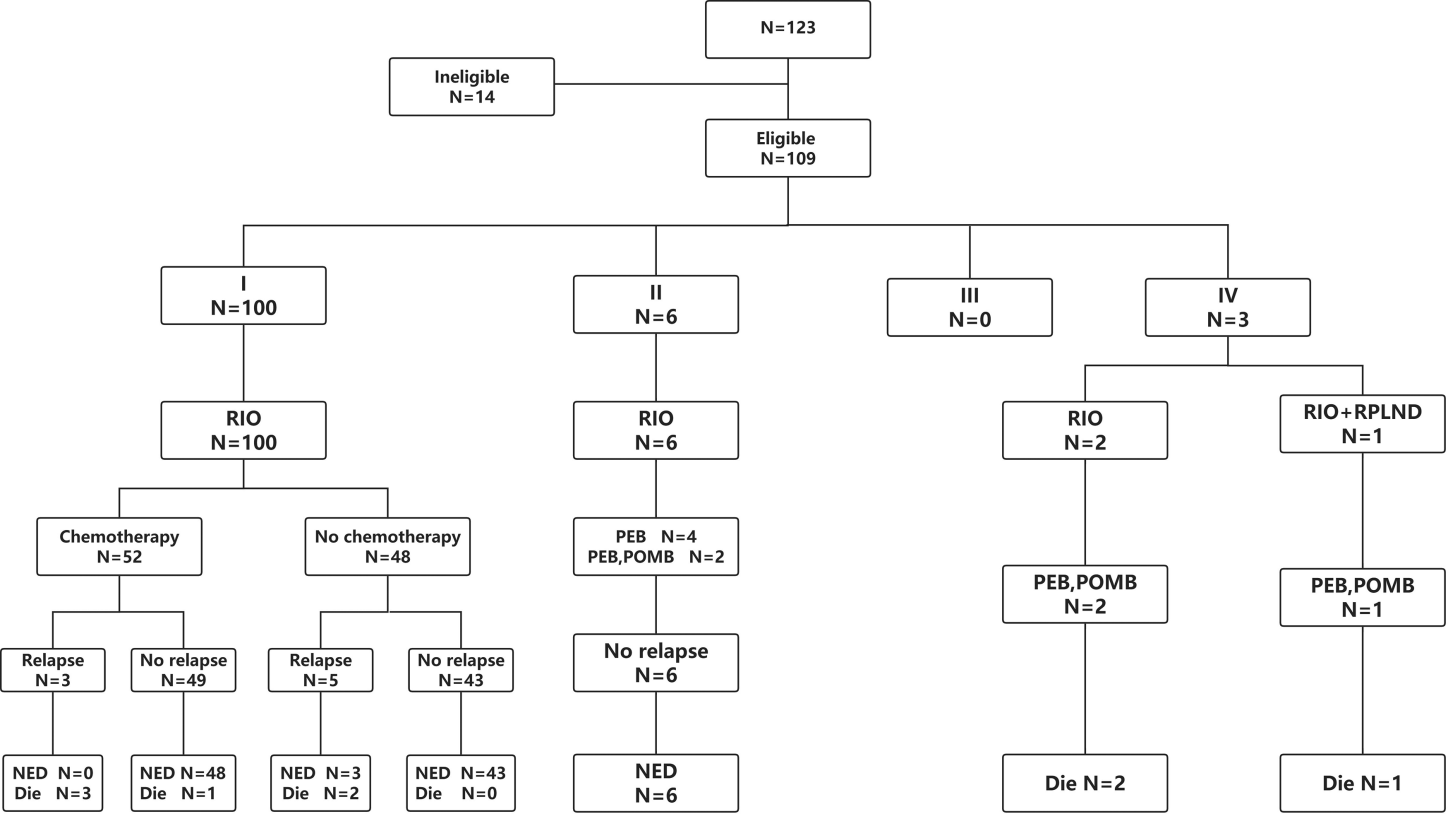
Study flow diagram. RIO, radical inguinal orchiectomy; RPLND, retroperitoneal lymph node dissection; PEB, cisplatin, etoposide and bleomycin; POMB, vincristine, cisplatin, methotrexate and bleomycin; NED, no existence of disease.

A total of 123 patients with TYST were treated at our hospital. One patient was diagnosed with a yolk sac tumor with teratoma, 4 patients underwent their first surgery at another hospital, and the clinical data of 9 patients were incomplete. Therefore, this study ultimately included 109 (88.6%) patients (Figure 1). The median age at diagnosis was 18 months (range 2–69). The numbers of patients aged <1, 1–2, 2–3 and >3 years were 36 (33.1%), 45 (41.3%), 20 (18.3%) and 8 (7.3%), respectively. The mean time from initial clinical presentation to diagnosis was 2.8 months (ranging from 3 days to 19 months), with 56 cases on the left side and 53 on the right. The most common clinical manifestation was a painless testicular mass in 106 (97.2%) patients, followed by a painful testicular mass in 3 patients. Three cases were diagnosed with concomitant hydrocele/inguinal hernia, two with cryptorchidism, two with testicular epididymitis, one with testicular torsion and one with varicocele. One hundred patients were diagnosed with stage I COG, 6 with stage II, and 3 with stage IV. There was a correlation between the time of diagnosis and stage of COG (*R* = 0.249, *P* = 0.009). The clinical characteristics of the patients are shown in Table 1.

**Table 1:** Clinical characteristics of patients with TYST.

	All	Die	Survival	
	*N* = 109	*N* = 9	*N* = 100	*p*
Age	19.3 (11.6)	22.2 (6.65)	19.1 (12.0)	0.23
Clinical time*	2.80 (3.97)	4.09 (4.94)	2.68 (3.88)	0.43
Follow-up time	61.3 (54.3)	9.11 (3.14)	66.0 (54.2)	<0.001
Side:				1.0
Left	53 (48.6%)	4 (44.4%)	49 (49.0%)	
Right	56 (51.4%)	5 (55.6%)	51 (51.0%)	
COG stage				0.001
I	100 (91.7%)	6 (66.7%)	94 (94.0%)	
II	6 (5.5%)	0 (0.00%)	6 (6.00%)	
IV	3 (2.8%)	3 (33.3%)	0 (0.00%)	
Maximum diameter of tumor (cm)	3.28 (1.56)	5.13 (2.58)	3.12 (1.33)	0.048
US properties:				0.47
Solid	86 (78.9%)	6 (66.7%)	80 (80.0%)	
Cystic	2 (1.8%)	0 (0.00%)	2 (2.00%)	
Cystic solid	21 (19.3%)	3 (33.3%)	18 (18.0%)	
US blood flow:				1.0
High	100 (91.74%)	9 (100%)	91 (91.0%)	
Normal	8 (7.34%)	0 (0.00%)	8 (8.0%)	
Low	1 (0.92%)	0 (0.00%)	1 (1.0%)	
Chemotherapy:				0.29
No	48 (44.0%)	2 (22.2%)	46 (46.0%)	
Yes	61 (56.0%)	7 (77.8%)	54 (54.0%)	
Side effect of chemotherapy				0.074
No	96 (88.1%)	6 (66.7%)	90 (90.0%)	
Yes	13 (11.9%)	3 (33.3%)	10 (10.0%)	
Preoperative AFP:				1.000
<363 ng/ml	19 (17.4%)	1 (11.1%)	18 (18.0%)	
>363 ng/ml	90 (82.6%)	8 (88.9%)	82 (82.0%)	
AFP1*:				0.17
>10 ng/ml	58 (53.2%)	7 (77.8%)	51 (51.0%)	
<10 ng/ml	51 (46.8%)	2 (22.2%)	49 (49.0%)	
AFP2*:				<0.001
>10 ng/ml	17 (15.6%)	6 (66.7%)	11 (11.0%)	
<10 ng/ml	92 (84.4%)	3 (33.3%)	89 (89.0%)	
Relapse				<0.001
No	101 (92.7%)	4 (44.4%)	97 (97%)	
Yes	8 (7.34%)	5 (55.6%)	3 (3.0%)	

Clinical time*: time of initial clinical presentation to diagnosis; AFP1*: postoperative AFP within 1 month; AFP2*: postoperative AFP within 2 months.

### Serum AFP

Among the 109 patients, 1 case with normal AFP and 108 patients with elevated AFP levels preoperatively. Among them, 95 had AFP > 363 ng/ml, 10 had AFP levels between 100 and 363 ng/ml, 3 had AFP levels between 10 and 100 ng/ml. During the follow-up, the AFP data returned to normal within one month for 47.22% (51/108) of patients and within two months for 85.19% (92/108) of patients.

### Ultrasound

The maximum diameter of the testicular tumor was 3.28 cm(range 0.72–9.4). There were correlations between the maximum tumor diameter and stage of COG, time of postoperative AFP recovery, and survival (R = 0.452, −0.224 and −0.357, *P* < 0.05). Ultrasonography showed a solid mass in 86 patients (79.9%), solid-cystic mixed echoic mass in 21 patients (19.3%) and cystic mass in 2 patients (1.8%). Colour Doppler flow imaging showed rich blood flow inside and around the mass in 91.74% of patients (100/109), normal blood flow in 7.34% of patients (8/109), and low blood flow in 0.92% of patients (1/109).

### Surgery and chemotherapy

Radical inguinal orchiectomy (RIO) was performed in 108 patients, and RIO and retroperitoneal lymph node dissection (RPLND) were performed in 1 patient. Fifty-two patients in stage I and all patients in stage II and IV received chemotherapy. Since no clear guidelines were given before 2009, VD (vincristine and rapamycin D) or JEP (carboplatin, etoposide and bleomycin) was used as the chemotherapy regimen for patients with stage I, while PEB (cisplatin, etoposide and bleomycin) was used as the chemotherapy regimen during 2009–2019. For patients with stage II-IV disease, PEB was used as a chemotherapy regimen, and POMB (vincristine, cisplatin, methotrexate, bleomycin) was added to strengthen chemotherapy if necessary. Among the chemotherapy-treated patients, 13 suffered the side effects of adjuvant chemotherapy.

### Follow-UP

The mean follow-up time was 61.3 months (range 3–259). Relapse occurred in 3 out of 52 patients in stage I with chemotherapy, compared with 5 out of 48 patients without chemotherapy, with a median of 5 months (range 3–7). Of the 8 patients who relapsed, three had lung metastasis, two had retroperitoneal and lung metastases, two had retroperitoneal, kidney and lung metastases, and one had retroperitoneal, lung and peri-aortic dissemination. Four patients with relapse received salvage chemotherapy, and the others discontinued salvage therapy. In total, 100 patients survived, and 9 patients died (Table 2). The long-term survival outcomes between patients in stage I with and without chemotherapy were not different (*t* = 1.497, *P* = 0.137). The five-year event-free survival and overall survival rates were 87.1% (95% CI 80.4%–94.3%) and 90.6% (95% CI 84.6%–96.7%), respectively.

**Table 2:** Clinical characteristics of the 9 death patients with testicular yolk sac tumor.

Case no.	Age (months)	COG stage	Tumor diameter (cm)	Pre-AFP (ng/ml)	surgery	Adjuvant treatment	Post-AFP (yes/no)	Metastasis	Recurrence*	Follow-up (months)	Outcome	Reason of death
1	>24	I	3.9	>363	RIO	PEB	Yes	/	RL,Lung,Kidney	11	Die	Give up
2	0–24	I	3.3	>363	RIO	JEB	Yes	/	RL,Lung	9	Die	Give up
3	0–24	I	2.6	>363	RIO	PEB	Yes	/	RL, PAD,Lung	8	Die	Tumor progression
4	>24	IV	9.2	>363	RIO	RS + RPLND,PEB, POMB	No	RL, PAD, Lung	/	15	Die	Give up
5	0–24	I	2.6	<363	RIO	PEB	Yes	/	/	4	Die	Hepatic failure
6	>24	IV	5.7	>363	RIO	PEB, POMB	No	RL,Lung,Kidney,Pleura	/	6	Die	Give up
7	>24	IV	8	>363	RIO	PEB, POMB	No	RL,Lung, Liver	/	9	Die	Tumor progression
8	0–24	I	3.2	>363	RIO	/	No	/	RL,Lung	11	Die	Tumor progression
9	0–24	I	7.7	>363	RIO	/	No	/	RL,Lung,kidney	5	Die	Give up

Pre-AFP, preoperative AFP; Post-AFP, postoperative AFP returned to normal within 2 month; RIO, radical inguinal orchiectomy; RS + RPLND, Retroperitoneal tumor resection, retroperitoneal lymph node dissection; PEB, cisplatin, etoposide and bleomycin; JEB, carboplatin, etoposide and bleomycin; POMB,vincristine, cisplatin, methotrexate and bleomycin; RL, Retroperitoneal metastasis; RPLN, Retroperitoneal lymph node metastasis; PAD, peri-aortic dissemination; Recurrence*, recurrence with distant metastasis.

We found a correlation between relapse and AFP failure returning to normal within 2 months (*R* = 0.267, *P *< 0.001). We used univariate and multivariate Cox risk proportional regression models to analyse the associated factors affecting pediatric survival and recorded the risk ratio. Univariate Cox proportional hazards regression models showed that disease stage, relapse, maximum tumor diameter, and AFP returning to normal within 2 months postoperatively were risk factors affecting patient survival. Multivariate Cox regression analysis showed that higher disease stage and relapse were independent risk factors affecting patient survival. The results of the Cox regression analysis are shown in Table 3.

**Table 3:** Univariate and multivariate analyses of factors affecting pediatric TYST survival.

	Univariate			Multivariate		
	HR	95% CI	*P*	HR	95% CI	*P*
Age	1.03	0.98–1.09	0.286			
Clinical time*	1.06	0.94–1.21	0.333			
Side	1.30	0.35–4.84	0.697			
COG stage	25.43	6.25–103.52	<0.001	148.297	15.193	<0.001
Maximum diameter of tumor (cm)	1.48	1.16–1.88	0.001			
Ultrasonography*	2.33	0.58–9.31	0.232			
Chemotherapy	2.73	0.57–13.15	0.21			
Side effect of chemotherapeutic	3.90	0.97–15.6	0.054			
Preoperative AFP	1.69	0.21–13.5	0.621			
AFP 1*	0.28	0.06–1.33	0.109			
AFP 2*	0.08	0.02–0.32	<0.001			
Relapse	26.43	6.7–104.21	<0.001	94.575	10.624	<0.001

Clinical time*, time of initial clinical presentation to diagnosis; Ultrasonography*, property of testicular mass finding by ultrasound; AFP1*, AFP returned to normal within 1 month; AFP2*, AFP returned to normal within 2 months.

## Discussion

Compared with testicular tumors in adults, pediatric TYST, with a low incidence, has different and unique characteristics (8). Although TYST is the most common pediatric malignant germ cell tumor, there are few reports (5). It is crucial for pediatric surgeons and urologists to have clear management strategies and prognoses for TYST. To the best of our knowledge, this is the largest series of pediatric TYST in China and also one of the largest studies of pediatric TYST ever reported in the world (1, 2, 8, 9, 11).

TYST demonstrates a distinct distribution with the peak presenting at a median age of two years (8, 12). Our study population exactly mirrors the distribution of disease described; the median age at diagnosis was 18 months, and 74.3% of the patients had a peak incidence between 0 and 2 years. Previous studies showed that more than 90% of pediatric patients with testicular tumors presented with a hard painless testicular mass (13), and this figure was 97.2% in our study. In total, 8.3% of patients were diagnosed with concomitant hydrocele/inguinal hernia, cryptorchidism, testicular epididymitis, testicular torsion and varicocele. These results showed that patients with the above diseases might be misdiagnosed, so careful physical examination and ultrasound might be conducive to the early detection of testicular tumor and timely treatment (12).

Serum AFP is an essential marker for diagnosing and treating TYST (8). Yolk sac tumors were associated with elevated AFP levels, and elevations were seen in more than 90% of the patients, as shown in a previous study (9); such elevations were observed in 99.1% of the patients in this study. AFP may increase within the first year of life, and teratomas can also produce AFP. Serum AFP > 100 ng/ml was consistently strongly correlated with yolk sac tumor (2, 5). This is in keeping with the present study where 96.3% of patients had AFP > 100 ng/ml preoperatively. AFP is also an important indicator for follow-up. The biological half-life of AFP is 5 days; AFP should be measured again postoperatively to evaluate an appropriate decrease in levels (5). A previous study showed that patients with slowly declining postoperative AFP might tend to have a poor outcome (14). The level of AFP failed to return to normal in 52.78% of patients within 1 month postoperatively and 14.81% of patients within 2 months postoperatively in the present study. AFP levels failing to return to normal within 2 months postoperatively was correlated with relapse postoperatively. In addition, AFP levels failing to return to normal within 2 months postoperatively was an adverse factor for long-term survival. We infer that patients with AFP levels that fail to return to normal within 2 months postoperatively might be more likely to experience relapse, indicating a poor prognosis. Identifying patients at high risk of relapse might allow a more accurate prognosis prediction. Therefore, we suggest that timely interventions, such as chemotherapy in 1 to 2 cycles (6, 15), for patients with AFP levels that fail to return to normal within 2 months might improve the prognosis and mitigate the toxicities associated with salvage chemotherapy.

Colour Doppler ultrasound is the primary imaging technique preferred for patients with testicular tumors, and its sensitivity is almost 100% (8, 16, 17). Ultrasound cannot completely distinguish between benign and malignant tumors, and its sensitivity to metastasis is limited. CT scans of the chest, abdomen and pelvic cavity are recommended if there is a suspicion of metastasis (5, 6). The typical appearance of a malignant testicular mass is a solid, hypoechoic, and homogeneous mass from within the testis by ultrasonography (12), with CDFI showing increased blood flow in the mass (4). The present results were similar to those of previous studies. Based on ultrasonography, the case distribution for solid and solid-cystic mixed was 79.8% and 19.3%, respectively. Furthermore, 91.7% of patients had abundant blood flow. Additionally, the above ultrasound findings were not risk factors for prognosis in the present study. Previous studies found that the maximum diameter of testicular tumor was related to prognosis. In this study, the results showed that the maximum diameter of the tumor was one of the adverse risk factors for survival, which might indicate that the larger the tumor is, the worse the prognosis might be. In addition, there was a significant positive correlation between maximum tumor diameter and disease stage. Therefore, we suggest that patients with large tumor diameters should undergo a CT scan to assess the stage of the disease.

In the present study, 91.7% of patients were in stage I, which corresponded with previous studies reporting that 80%–90% of testicular yolk sac patients were in stage I (9, 12). Several studies have shown that adjuvant chemotherapy postoperatively is necessary for patients with stage I disease, while COG protocols propose surveillance in those after RIO (5). Our study showed no difference in survival rates for patients with stage I disease between those with and without chemotherapy; the current findings agree with Rescola et al. (18). Platinum-based regimens have several serious side effects for pediatric patients (19–21). One patient with stage I died from the side effects of chemotherapy in our study; thus, we recommend patients with stage I may avoid unnecessary chemotherapy after RIO, and close surveillance is necessary during the follow-up, which is helpful to avoid excessive treatment. Eight patients with stage I experienced recurrence within the first year after RIO, while 4 cases of them gave up therapy. Overall, the survival rate of patients with stage I disease was 94.0%. Patients with stage II received adjuvant chemotherapy after RIO; they all survived at the end of the study. There were no cases of stage III in our study, perhaps because these cases are rare, which was similar to the previous study (1). All patients in stage IV, with a median survival time of 9 months, ultimately died. In the present study, the five-year overall survival rate was 90.6%, consistent with that of Koehne et al. (11).

Due to the low incidence, the prognostic factors of TYST are still unclear. Although several previous studies showed that maximum tumor diameter and histological type were adverse prognostic factors for patients with TYST (22, 23), they were not independent adverse prognostic factors in this study. Our study showed that a higher disease stage and relapse were independent risk factors for an adverse prognosis. A higher disease stage may mean a worse prognosis; therefore, our results agree with Frazier et al.'s study (23). Tumor recurrence and metastasis are adverse factors for the survival outcomes of patients with TYST. Liu et al. reported that 23.8% of cases with stage I disease experienced relapse, while the survival rate was still high after salvage chemotherapy. In the present study, 8.0% of patients with stage I experienced relapse, and the survival rate of recurrent metastases was only 37.5%, which is lower than that reported in previous literature. This may be due to various reasons, such as economic factors and parental education. 50% (4/8) of patients with recurrence gave up salvage chemotherapy; however, salvage chemotherapy was effective for 75% (3/4) of patients. The present relapse rate was significantly lower than that in previous studies (9). Adolescent patients were always included in previous studies, while there were no adolescent cases in the present study. No relapse was found in the study of O'Shea et al., whose study cohort, all yolk sac tumors were seen in the prepubertal age group with stage I diseases were cured by RIO (2). Based on this, we inferred that age might be a risk factor affecting recurrence, and that the relapse rate of younger patients with stage I disease might be lower than that of older patients, which should be explored in future clinical studies. In addition, we were surprised to find relapse patients with only lung metastasis who survived from salvage chemotherapy in this study. However, the number of patients was small, and the effectiveness needs further exploration.

The current study has certain limitations. First, this was a retrospective study, which might be subject to selection and recall biases, even though the lost visit rate was low. Second, some patients underwent several examinations in other hospitals before surgery, which resulted in losing some image data in our record system. Third, education was needed for patients and parents in China since four relapsed patients gave up salvage treatment due to a lack of knowledge about the disease. However, we believe that our results can enhance our knowledge about this rare tumor.

## Conclusion

Our results suggest that the prognosis of pediatric TYST is generally excellent.TYST had a peak incidence in children aged <2 years. A painless mass was the most common presentation, and 99.1% of patients had preoperative AFP elevation. Nearly 92% of the patients were in stage I, and relapse mainly occurred during the first year postoperatively. A higher disease stage and relapse predicted a poor prognosis. Individualized management of children with TYST according to risk classification is feasible.

## Data Availability

The original contributions presented in the study are included in the article/Supplementary files, further inquiries can be directed to the corresponding author/s.
